# In-depth characterization of multidrug-resistant NDM-1 and KPC-3 co-producing *Klebsiella pneumoniae* bloodstream isolates from Italian hospital patients

**DOI:** 10.1128/spectrum.03305-23

**Published:** 2024-02-27

**Authors:** Brunella Posteraro, Flavio De Maio, Yair Motro, Giulia Menchinelli, Desy De Lorenzis, Roberto B. M. Marano, Bessan Aljanazreh, Federica Maria Errico, Giuseppe Massaria, Teresa Spanu, Patrizia Posteraro, Jacob Moran-Gilad, Maurizio Sanguinetti

**Affiliations:** 1 Dipartimento di Scienze Biotecnologiche di Base, Cliniche Intensivologiche e Perioperatorie, Università Cattolica del Sacro Cuore, Rome, Italy; 2 Dipartimento di Scienze Mediche e Chirurgiche Addominali ed Endocrino Metaboliche, Fondazione Policlinico Universitario A. Gemelli IRCCS, Rome, Italy; 3 Dipartimento di Scienze di Laboratorio e Infettivologiche, Fondazione Policlinico Universitario A. Gemelli IRCCS, Rome, Italy; 4 Department of Health Policy and Management, School of Public Health, Faculty of Health Sciences, Ben-Gurion University of the Negev, Beer-Sheva, Israel; 5 GVM - Ospedale San Carlo di Nancy, Laboratorio di Analisi Chimico-Cliniche e Microbiologiche, Rome, Italy; 6 GVM - Ospedale San Carlo di Nancy, Direzione Sanitaria, Rome, Italy; Laboratory Corporation of America Holdings, Burlington, USA

**Keywords:** *Klebsiella pneumoniae*, *bla*_NDM-1_, *bla*_KPC-3_, carbapenemase-producing, antimicrobial resistance, whole-genome sequencing

## Abstract

Bloodstream infection (BSI) caused by carbapenem-resistant *Klebsiella pneumoniae* (KP) poses significant challenges, particularly when the infecting isolate carries multiple antimicrobial resistance (AMR) genes/determinants. This study, employing short- and long-read whole-genome sequencing, characterizes six New Delhi metallo-β-lactamase (NDM) 1 and KP carbapenemase (KPC) 3 co-producing KP isolates, the largest cohort investigated in Europe to date. Five [sequence type (ST) 512] and one (ST11) isolates were recovered from patients who developed BSI from February to August 2022 or February 2023 at two different hospitals in Rome, Italy. Phylogenetic analysis revealed two distinct clusters among ST512 isolates and a separate cluster for the ST11 isolate. Beyond *bla*_NDM-1_ and *bla*_KPC-3_, various AMR genes, indicative of a multidrug resistance phenotype, including colistin resistance, were found. Each cluster-representative ST512 isolate harbored a *bla*_NDM-1_ plasmid (IncC) and a *bla*_KPC-3_ plasmid [IncFIB(pQil)/IncFII(K)], while the ST11 isolate harbored a *bla*_NDM-1_ plasmid [IncFII(pKPX1)] and a *bla*_KPC-3_ plasmid [IncFIB(K)/IncFII(K)]. The *bla*_NDM-1_ plasmids carried genes conferring resistance to clinically relevant antimicrobial agents, and the aminoglycoside resistance gene *aac*(*6*′)-*Ib* was found on different plasmids. Colistin resistance-associated *mgrB*/*pmrB* gene mutations were present in all isolates, and the yersiniabactin-encoding *ybt* gene was unique to the ST11 isolate. In conclusion, our findings provide insights into the genomic context of *bla*_NDM-1_/*bla*_KPC-3_ carbapenemase-producing KP isolates.

**IMPORTANCE:**

This study underscores the critical role of genomic surveillance as a proactive measure to restrict the spread of carbapenemase-producing KP isolates, especially when key antimicrobial resistance genes, such as *bla*_NDM-1_/*bla*_KPC-3_, are plasmid borne. In-depth characterization of these isolates may help identify plasmid similarities contributing to their intra-hospital/inter-hospital adaptation and transmission. Despite the lack of data on patient movements, it is possible that carbapenem-resistant isolates were selected to co-produce KP carbapenemase and New Delhi metallo-β-lactamase via plasmid acquisition. Studies employing long-read whole-genome sequencing should be encouraged to address the emergence of KP clones with converging phenotypes of virulence and resistance to last-resort antimicrobial agents.

## INTRODUCTION

Carbapenem-resistant *Klebsiella pneumoniae* (KP) bloodstream infection (BSI) has become a global public health threat, particularly in neonatal and intensive care hospital settings (1–3). Antimicrobial resistance (AMR) limits therapeutic options to treat KP-BSI (4, 5), which is, not surprisingly, associated with high mortality rates (6, 7). While intestinal colonization by or environmental exposure to KP can lead to bacteremia (8), the EuSCAPE study showed that KP isolates harboring carbapenemases have a higher capacity transmission (intra-hospital or inter-hospital within same countries) compared to those not harboring carbapenemases (9). The most common KP carbapenemase (KPC) types, namely, KPC-2 and KPC-3, are encoded by the plasmid-borne genes *bla*_KPC-2_ and *bla*_KPC-3_, respectively (10). According to recent data (8), *bla*_KPC-2_ and *bla*_KPC-3_ were found in Italy in 10% or 90% of carbapenem-resistant isolates from patients with BSI, respectively. These isolates mostly belong to the clonal group (CG) 258 (CG258), which includes the sequence type (ST) 11 (ST11) and ST258/512 as major phylogenetic lineages or clones (i.e., identified through allelic variation in core genes) (9, 11). Notably, ST258 isolates are hybrid isolates, originating from a large recombinational replacement event between the chromosomes of ST11 (with which they share ~80% of homology) and ST442-like (with which they share ~20% of homology) isolates (12).

In 2018, the draft genome sequence of a clinical KP isolate from a Chinese hospital (strain NUHL30457) co-producing New Delhi metallo-β-lactamase (NDM) 1 and KPC-2 was published (13). Since then, KP organisms carrying multiple (NDM and KPC) carbapenemase genes have emerged in China and in other world regions (14, 15). Although both NDM- and KPC-type carbapenemases confer high-level resistance to carbapenems, their coexistence with other AMR mechanisms [e.g., mediated by extended-spectrum β-lactamase (ESβL) or aminoglycoside-modifying enzymes] or with virulence determinants (e.g., the siderophore yersiniabactin) makes treatment of KP-BSI more challenging (16). Multidrug-resistant (MDR) KP clones (CG258, CG307, etc.) were distinguished from hypervirulent KP clones (CG23, CG65, etc.) (17), until convergent KP clones emerged, consisting of organisms with both MDR and hypervirulence phenotypes (18). Unlike KPC-2 and KPC-3, NDM-1 along with other metallo-β-lactamases (MβLs, e.g., IMP-1 or VIM-1) are not inhibited by avibactam (which is used in combination with ceftazidime) (19).

In a 5-year genomic surveillance study involving public hospitals in Singapore, plasmid-mediated transmission accounted for ~50% of dissemination of carbapenemase-producing KP (and other *Enterobacterales*), whereas another ~40% met clonal (i.e., clone mediated) transmission criteria (20). Regarding KP, whole-genome sequencing (WGS) is the most cost-efficient approach to deeply characterize clinical isolates (16), and bioinformatic tools facilitate the analysis and interpretation of genomic data (21). Here, we reported on the WGS-based characterization of KP isolates co-producing NDM-1 and KPC-3 from hospitalized patients in Italy.

## RESULTS

### Phenotypic determination of AMR in study isolates

We studied NDM-1 and KPC-3 co-producing KP isolates, five (CRKP2201 to CRKP2205) and one (BSI_389-23) of which were obtained from patients with BSI at two respective hospitals in Rome, Italy. Based on *in vitro* antimicrobial susceptibility testing results (Table S1 in the supplemental material), isolates showed phenotypic resistance to carbapenems, expanded-spectrum cephalosporins (e.g., third-generation cephalosporins), the β-lactam/β-lactamase inhibitor combinations ceftazidime-avibactam (CAZavi) and ceftolozane-tazobactam, fluoroquinolones, aminoglycosides, and trimethoprim-sulfamethoxazole. Isolates were also resistant to colistin and, excluding BSI_389-23, to cefiderocol. Accordingly, isolates displayed an MDR phenotype, i.e., they were non-susceptible to ≥1 agent in ≥3 antimicrobial classes (22). Isolates were not tested for susceptibility to fosfomycin, sulphonamides, phenicols (e.g., chloramphenicol), or macrolides (e.g., erythromycin).

### Phylogenetic analysis of study isolates

**Fig 1:**
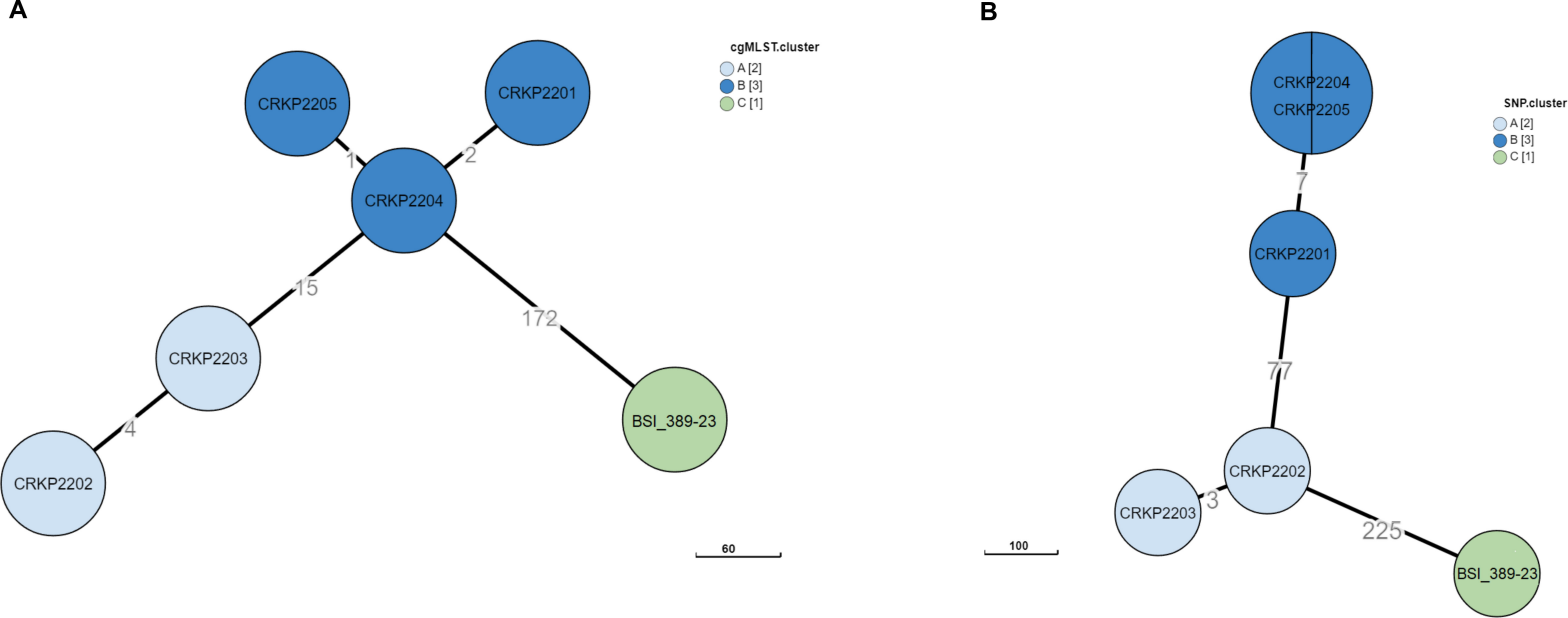
Phylogenetic trees showing differences between the six KP isolates from patients with BSI at two hospitals in Italy. The minimum spanning trees were created based on cgMLST (panel **A**) or core genome SNPs (panel **B**). In each tree, nodes are denoted as circles, with different colors indicating the three clusters formed by isolates. Except for one (larger) node (panel **B**), which includes two isolates, each of (remaining) nodes represents a single isolate. Numbers indicate allele (panel **A**) or SNP (panel **B**) differences between nodes. In both trees, isolates in cluster A (CRKP2202 and CRKP2203) or cluster B (CRKP2201, CRKP2204, and CRKP2205) differed markedly from the isolate in cluster C (BSI_389-23).

According to the short read (SR)-based multilocus sequence typing (MLST) analysis (seven loci), five isolates (CRKP2201 to CRKP2205) were classified as ST512, and one isolate (BSI_389-23) was classified as ST11. As depicted in Fig. 1A, *ad hoc* core genome MLST (cgMLST) analysis (864 loci) grouped the five isolates separately from BSI_389-23 (month-day-year, 02-23-2023), while consisting of two clusters. One cluster was formed by isolates CRKP2201 (02-14-2022), CRKP2204 (06-02-2022), and CRKP2205 (08-25-2022); and the other cluster, by isolates CRKP2202 (03-28-2022) and CRKP2203 (04-20-2022). The three isolates differed from the other two isolates by 15 loci; and from BSI_389-23, by 172 loci. After mapping SR sequences to the BSI_389-23 hybrid assembly, a recombination-free core-genome single-nucleotide polymorphism (SNP)-based tree was created to assess the phylogenetic relationship of the six isolates. As depicted in Fig. 1B, CRKP2204 and CRKP2205 were related to CRKP2201 (seven-SNP distance) but were distant from both CRKP2202 and CRKP2203 (by 84 SNPs) and, as expected, from BSI_389-23 (by 309 SNPs). The isolates CRKP2202 and CRKP2203 differed from each other by only three SNPs.

### Resistome profiling of study isolates

**Fig 2:**
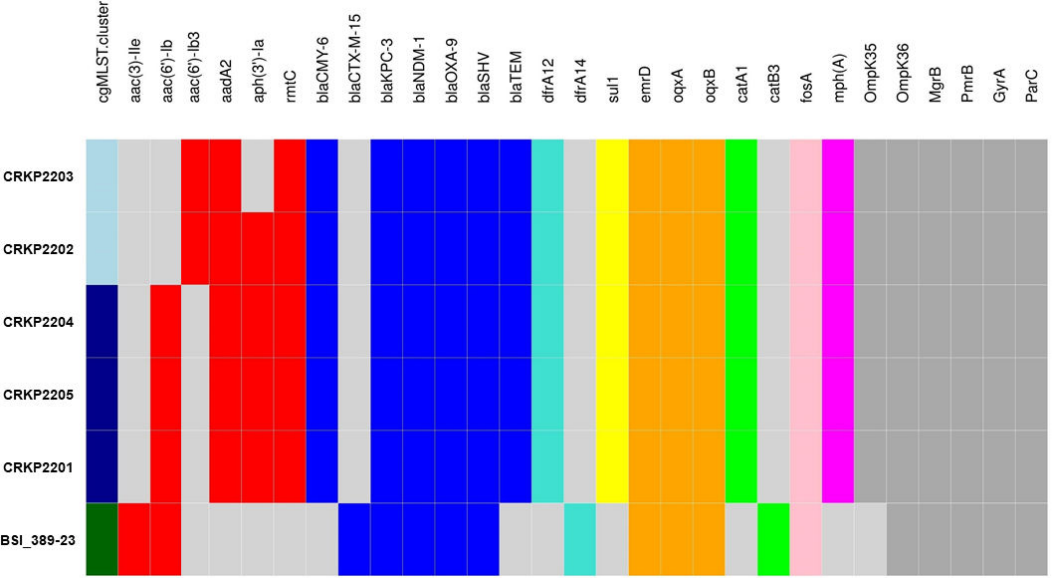
Resistome profiling for the six KP isolates in the study. A matrix of the distribution of AMR determinants (x axis) among the isolates within the indicated cgMLST clusters (A, light blue; B, dark blue; and C, green) is shown. According to different colors, blocks indicate the presence of an acquired gene (red, aminoglycoside resistance; blue, β-lactam resistance; turquoise, trimethoprim resistance; yellow, sulphonamide resistance; orange, fluoroquinolone resistance; green, phenicol resistance; pink, fosfomycin resistance; magenta, macrolide resistance) identified using AMRFinderPlus, the presence of a mutated/truncated gene (dark gray) identified using Kleborate, or the absence of any AMR determinant (light gray). Mutations included truncation of OmpK35 and GD insertion in the OmpK36 β-strand loop (both known to contribute to the carbapenem resistance phenotype), truncations in MgrB/PmrB (known to confer colistin resistance), and mutations in the quinolone resistance-determining region of GyrA (S83L) and ParC (S80I) (both known to confer fluoroquinolone resistance).

Figure 2 shows the SR-based resistome analysis, which revealed the presence of the putative chromosomal *fosA* (fosfomycin resistance) and *oqxA*/*oqxB* (fluoroquinolone resistance) genes as well as mutated/truncated genes, such as *ompK35*/*ompK36* (carbapenem resistance), *mgrB*/*pmrB* (colistin resistance), or *gyrA*/*parC* (fluoroquinolone resistance), in all six isolates. Regarding the putative acquired resistome, along with *bla*_NDM-1_ and *bla*_KPC-3_ genes, other β-lactam (*bla*_OXA-9_), trimethoprim (*dfrA12*/*dfrA14*), or phenicol (*catA1*/*catB3*) resistance genes, and at least one gene that confers aminoglycoside resistance [e.g., *aac*(*6*′)-*Ib*] were detected in all six isolates. The sulphonamide (*sul1*) or erythromycin (*mphA*) resistance genes were detected in all but BSI_389-23 isolates, whereas the β-lactam resistance gene *bla*_CTX-M-15_ was only detected in the BSI_389-23 isolate. The AMR determinants (mutations and/or acquired genes) identified were consistent with the above-described MDR phenotype for all six isolates. No CAZavi resistance-associated mutations (19) were found in the isolates’ *bla*_KPC-3_ genes, suggesting that co-production of a (less sensitive) NDM-type MβL could have caused the CAZavi resistance phenotype of isolates. No plasmid-borne colistin resistance gene *mcr-1* (23) was detected in any of the isolates, supporting the complexity of some AMR mechanisms in KP organisms.

### Plasmid characterization in cluster-representative isolates

The isolates CRKP2202 and CRKP2205 were selected along with BSI_389-23 for a long-read (LR) sequencing approach. Subsequent hybrid genome assemblies allowed us to assess the gene content, plasmid content, and AMR or virulence determinants for the three isolates, respectively. The number of annotated coding DNA sequences per genome was 5,484 for CRKP2202, 5,744 for CRKP2205, and 5,401 for BSI_389-23. Of 25 AMR genes detected in total (see also Fig. 2), five were in the chromosome, and 20 were in plasmids.

**Fig 3:**
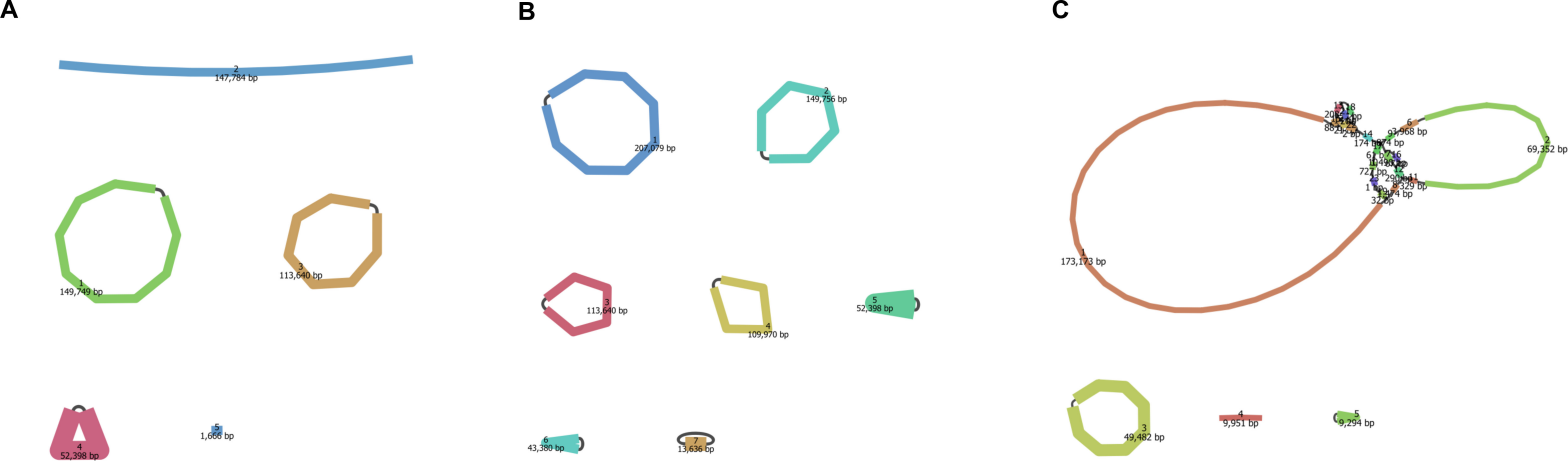
Bandage plots of the plasmids identified in the three KP isolates (CRKP2202, CRKP2205, and BSI_389-23) for which both long-read and short-read sequencing data were available. (**A**) In the CRKP2202 isolate, three of five plasmids (size range, 1,666–149,749 bp) were circular, and two were non-circular. (**B**) In the CRKP2205 isolate, seven of seven plasmids (size range, 13,636–207,079 bp) were circular. (**C**) In the BSI_389-235 isolate, four of eight plasmids (size range, 727–173,173 bp) were circular, and four were non-circular.

Figure 3 depicts the structure of the plasmids identified in CRKP2202, CRKP2205, and BSI_389-23, and Table 1 summarizes their structural and non-structural characteristics, including size, replicon type, and harbored AMR gene(s). The largest plasmid (207,079 bp), an IncFIB(K)/IncFII(K) plasmid, was found in CRKP2205, and seven different plasmids based on the type(s) of replicons were identified among the three isolates. The *bla*_NDM-1_ gene was harbored by an IncC plasmid in both CRKP2202 and CRKP2205 and by an IncFII(pKPX1) plasmid in the BSI_389-23 isolate. Despite their different genetic context, the two plasmids shared a large array of AMR genes (six and eight, respectively), which also included *bla*_CMY-6_ in one case (IncC plasmid) or both *bla*_CTX-M-15_ and *bla*_OXA-1_ in the other case [IncFII(pKPX1) plasmid]. The *bla*_KPC-3_ gene was harbored by an IncFIB(pQil)/IncFII(K) plasmid in both CRKP2202 and CRKP2205 and by an IncFIB(K)/IncFII(K) plasmid in the BSI_389-23 isolate. Conversely, only two other genes (*bla*_OXA-9_ and *bla*_TEM-1_) were in the IncFIB(pQil)/IncFII(K) plasmid, and no other genes were in the IncFIB(K)/IncFII(K) plasmid. In CRKP2205, two plasmids, an IncX3 plasmid and a ColRNAI plasmid, harbored only one gene [*bla*_SHV-11_ and *aac*(*6*′)-*Ib*, respectively]. Notably, *aac*(*6*′)-*Ib* and *sul1* were found in three different plasmids [IncC, ColRNAI, and IncFII(pKPX1) or IncC, IncFIB(K), and IncFIB(K)/IncFII(K), respectively]. No AMR genes were found in a IncFIB(pKPHS1) plasmid from the CRKP2205 isolate or in a ColRNAI plasmid from the BSI_389-23 isolate.

**TABLE 1:** Summary of the characteristics of the plasmids identified in three *K. pneumoniae* isolates included in the study^*a*^

Genome assembly contig	Size (bp)	Replicon type	GenBank accession no.	Resistance gene(s) harbored by the plasmid
Isolate CRKP2202				
contig_1	149,749	IncC	JN157804	aac(6′)-Ib, bla_CMY-6_, bla_NDM-1_, ble, rmtC, sul1
contig_2	147,784	IncFIB(K)	JN233704	aadA2, aph(3′)-Ia, catA1, dfrA12, mph(A), sul1
contig_3	113,640	IncFIB(pQil)/IncFII(K)	JN233705/CP000648	bla_KPC-3_, bla_OXA-9_, bla_TEM-1_
contig_4	52,398	–	–	–
contig_5	1666	–	–	–
Isolate CRKP2205				
contig_1	207,079	IncFIB(K)/IncFII(K)	JN233704/CP000648	aadA2, aph(3′)-Ia, catA1, dfrA12, mph(A), sul1
contig_2	149,756	IncC	JN157804	aac(6′)-Ib, bla_CMY-6_, bla_NDM-1_, ble, rmtC, sul1
contig_3	113,640	IncFIB(pQil)/IncFII(K)	JN233705/CP000648	bla_KPC-3_, bla_OXA-9_, bla_TEM-1_
contig_4	109,970	IncFIB(pKPHS1)	CP003223	–
contig_5	52,398	–	–	–
contig_6	43,380	IncX3	JN247852	bla_SHV-11_
contig_7	13,636	ColRNAI	DQ298019	aac(6′)-Ib
Isolate BSI_389–23				
contig_1	173,173	IncFIB(K)/IncFII(K)	JN233704/CP000648	bla_KPC-3_
contig_2	69,352	IncFII(pKPX1)	AP012055	aac(3)-IIe, aac(6′)-Ib, bla_CTX-M-15_, bla_NDM-1_, bla_OXA-1_, ble, catB3, dfrA14
contig_3	49,482	–	–	–
contig_4	9951	–	–	–
contig_5	9294	ColRNAI	DQ298019	–
contig_6	3968	–	–	–
contig_7	1490	–	–	–
contig_10	727	–	–	–

^*a*^ "-" means the absence of data about the indicated characteristic.

**Fig 4:**
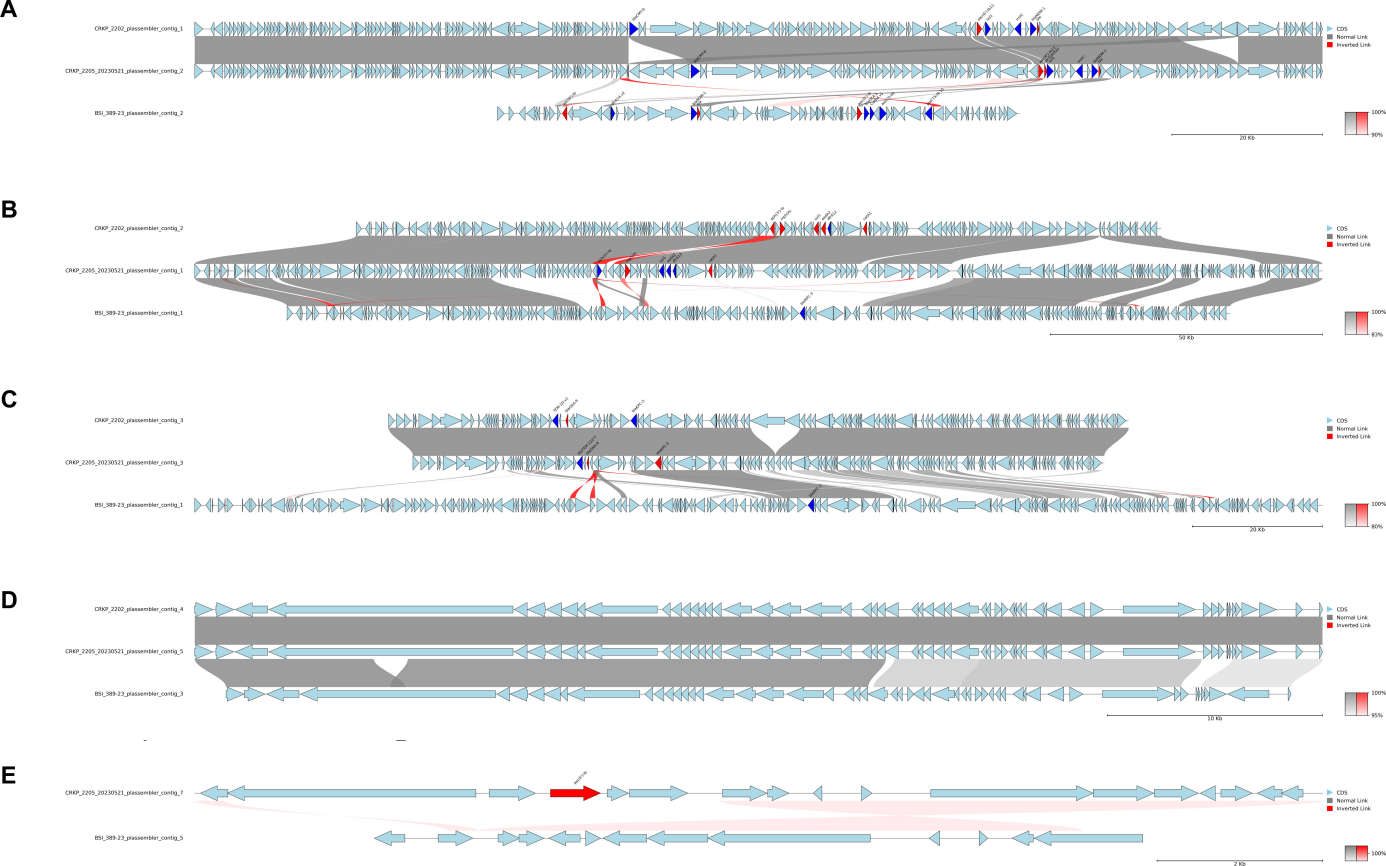
Comparison of the plasmids identified in the three KP isolates (CRKP2202, CRKP2205, and BSI_389-23) for which both long-read and short-read sequencing data were available. MUMmer-based sequence alignment was performed for (**A**) plasmids harboring *bla*_NDM-1_ (CRKP2202, contig_1; CRKP2205, contig_2; BSI_389-23, contig_2), (**B**) plasmids harboring AMR genes other than *bla*_NDM-1_ or *bla*_KPC-3_ (CRKP2202, contig_2; CRKP2205, contig_1; BSI_389-23, contig_1), (**C**) plasmids harboring *bla*_KPC-3_ (CRKP2202, contig_3; CRKP2205, contig_4; BSI_389-23, contig_1), (**D**) plasmids lacking AMR genes (CRKP2202, contig_4; CRKP2205, contig_5; BSI_389-23, contig_3), and (**E**) ColRNAI plasmids, with one of them harboring only the *aac*(*6*′)-*Ib* gene (CRKP2205, contig_7; BSI_389-23, contig_5). Blocks between plasmids indicate sequence identities of 90%–100% (**A**), 83%–100% (**B**), 80%–100% (**C**), 95%–100% (**D**), or 95%–100% (**E**), respectively.

To explore potential relationships between plasmids, a MUMmer-based comparative analysis of plasmids from CRKP2202, CRKP2205, and BSI_389–23 was performed. As depicted in Fig. 4, *bla*_NDM-1_-harboring plasmids (IncC) from CRKP2202 and CRKP2205 showed a highly conserved structure, which differed from that of the *bla*_NDM-1_-harboring plasmid [IncFII(pKPX1)] in BSI_389-23. Conversely, *bla*_KPC-3_-harboring plasmids [IncFIB(pQil)/IncFII(K)] in CRKP2202 and CRKP2205 showed notable similarity and shared highly conserved regions with the *bla*_KPC-3_-harboring plasmid [IncFIB(K)/IncFII(K)] in BSI_389-23. In CRK2202 and CRKP2205, plasmids [IncFIB(K)/IncFII(K)] harboring AMR genes other than *bla*_NDM-1_ had nearly identical regions, corresponding to the regions where the genes [*aadA2*, *aph(3′)-Ia*, *catA1*, *dfrA12*, *mph(A*), and *sul1*] were located. Notably, the two respective plasmids shared regions lacking AMR genes with the *bla*_KPC-3_-harboring plasmid of BSI_389-23 as well as an indel region, suggesting intermixing between several plasmids of the three isolates.

### Molecular determinants of colistin resistance or virulence

**Fig 5:**
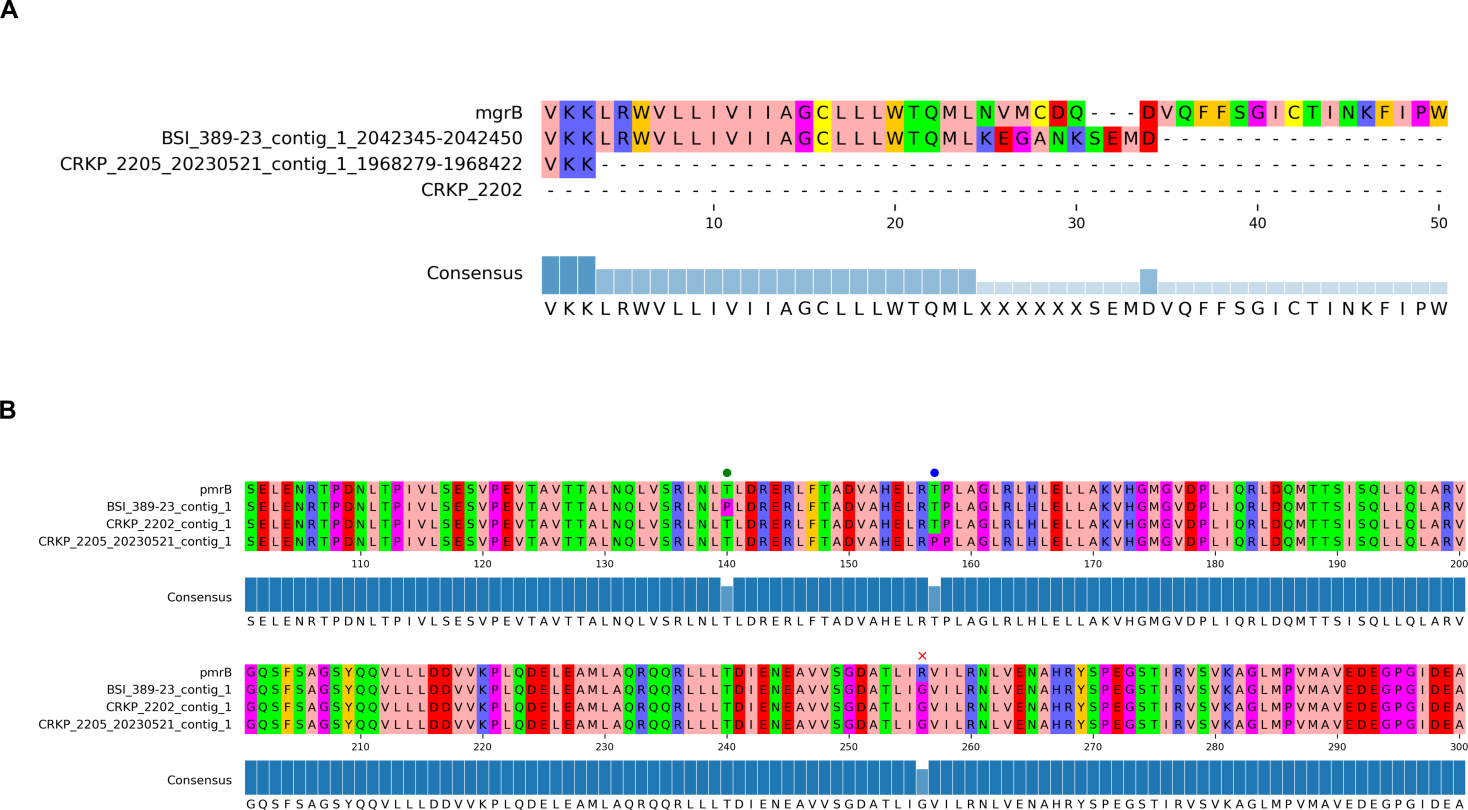
Schematic representation of colistin resistance molecular determinants in the three KP isolates (CRKP2202, CRKP2205, and BSI_389-23) for which both long-read and short-read sequencing data were available. Multiple amino acid sequence alignments of MgrB (**A**) and PmrB (**B**) from the three hybrid assemblies against the reference (Kleborate retrieved) wild type were performed. (**A**) MgrB from BSI_389-23 is truncated at amino acid position 34, MgrB from CRKP2205 is truncated at amino acid position 3, and MgrB is absent in CRKP2202. (**B**) PmrB from all three isolates showed the R256G mutation (denoted as a red x). Additionally, T140P (denoted as a green dot) or T157P (denoted as a blue dot) mutation was identified in BSI_389-23 and CRKP2205, respectively.

A detailed description of colistin resistance determinants in the three isolates (CRKP2202, CRKP2205, and BSI_389-23) is provided in Fig. 5. A Kleborate-based AMR analysis allowed us to search for missense mutations (alterations to the amino acid sequence), nonsense mutations (premature stop codon), or frameshift mutations (by insertions/deletions) in the *mgrB* and *pmrB* genes (23). As a result, we found that MgrB was absent in CRKP2202 (complete deletion) but was present in CRKP2205 (6% truncation) and BSI_389-23 (60% truncation; in this case, an insertion was followed by a stop codon). Regarding PmrB, all three isolates had the R256G mutation, whereas CRKP2205 also had the T157P mutation, and BSI_389-23 also had the T140P mutation.

Finally, a Kleborate-based virulome analysis revealed the presence of the *ybt* gene encoding the acquired yersiniabactin in the BSI_389-23 but not in the CRK2202 and CRKP2205 isolates. No other acquired virulence determinants, such as the siderophores aerobactin (*iuc*) and salmochelin (*iro*), the genotoxin colibactin (*clb*), and the hypermucoidy locus (*rmpADC*) (21), were detected.

## DISCUSSION

This study represents the first comprehensive WGS-based characterization of KP-BSI isolates from Italian hospital patients co-producing NDM-1 and KPC-3 carbapenemases. Beyond *bla*_NDM-1_ and *bla*_KPC-3_, the six isolates, five from clone ST512 (CRKP2201 to CRKP2205) and one from clone ST11 (BSI_389-23), exhibited an array of AMR genes and chromosomal mutations, indicative of an MDR phenotype observed *in vitro*. While isolates (five and one) formed clusters corresponding to the respective hospitals where BSIs were diagnosed, genomic similarities within ST512 or ST11 clusters and even between clusters highlighted shared AMR plasmids. This underscores that, although certain AMR genes and plasmids are linked to specific clones (ST512 or ST11) in hospital-acquired BSI isolates, some plasmid similarities extend beyond these clones and healthcare facilities, indicating broader dissemination.

### High-risk KP clones and clinical implications

The KP clones ST258 and its derivative ST512 are a cause of concern due to their spatiotemporal expansion in European hospitals, linked to nosocomial transmission and genomic detection of carbapenem resistance genes (9). Shropshire et al. (24) used hybrid SR and LR genome assemblies to dissect the accessory genome of carbapenem-resistant KP isolates (CG258 and co-circulating CG307) in Houston, TX, hospitals. CG307 isolates lacked siderophore genes, suggesting that convergence of MDR and hypervirulence molecular determinants may be a hallmark of CG258. The Shropshire et al.’s study (24) reported higher rates of BSI and 30-day mortality in CG258-infected patients compared to CG307-infected patients. In our study, all patients (5/5) patients with ST512 KP-BSI succumbed, highlighting the challenges in managing carbapenem-resistant (and MDR) KP isolates. Notably, the patient with ST11 KP-BSI, treated with cefiderocol (to which the isolate was susceptible *in vitro*), survived.

The KP clone ST11, derived from ST258 by recombination, differs geographically (17). The Shropshire et al.’s study (24) reported that 2 of 37 CG258 isolates were third-generation cephalosporin resistant due to a *bla*_CTX-M-15_-harboring plasmid and were carbapenem resistant due to a *bla*_KPC-3_-harboring plasmid. Only one isolate in our study (ST11) had a plasmid-borne *bla*_CTX-M-15_. These findings underscore the diversity of AMR genes within the same clone across locations, emphasizing the role of plasmid content in phenotypic resistance.

### Uncommon coexistence of multiple carbapenemases in KP-BSI isolates

Since the first identification of KPC in the USA in 1996, NDM-1 and other non-KPC-type carbapenemases, such as MβLs, have emerged but to a lesser extent (43 KPC variants versus 28 NDM variants) (10, 25). Alarmingly, NDM-1, especially in combination with KPC-2/KPC-3, is on the rise. In 2021, the Pan American Health Organization/World Health Organization warned of NDM/KPC combinations in *Enterobacterales*, including KP, in Latin America and the Caribbean (26). This may be linked to increased broad-spectrum antibiotic use in COVID-19 patients, impacting microbiological diagnosis and antimicrobial agent use. Gao et al. (14) studied seven carbapenem-resistant KP isolates in China from 2012 to 2017. They suggested that extensive CAZavi use [amplified by COVID-19; see reference (27)] may have counter-selected isolates with only *bla*_KPC-2/3_, while highly transferable *bla*_NDM-1_ plasmids contributed to intra-hospital/inter-hospital adaptation and transmission of isolates carrying both *bla*_KPC-2/3_ and *bla*_NDM-1_. In our study, similar clinical contexts could have selected for KPC-2/KPC-3 carbapenem-resistant KP isolates and induction of co-produced KPC and NDM via plasmid acquisition.

### Cluster-associated plasmids and AMR profiles

Our analysis of hybrid genome assemblies in three cluster-representative KP isolates (CRK2202, CRKP2205, and BSI_389-23) revealed that *bla*_NDM-1_ and *bla*_KPC-3_ were located on distinct but cluster-associated plasmids. The *bla*_NDM-1_ plasmids harbored genes conferring resistance to various antimicrobial agents, including aminoglycoside [*aac*(*6*′)-*Ib*)], trimethoprim (*dfrA14*), phenicol (*catB3*), bleomycin (*ble*), and sulphonamide (*sul1*). In contrast to BSI_389-23, CRK2202 and CRKP2205 carried an additional AMR plasmid that did not harbor *bla*_NDM-1_ or *bla*_KPC-3_. Further analysis showed that while the plasmids in CRK2202 and CRKP2205 were identical, they differed from those in BSI_389-23, not only in the number and type of AMR genes but also in the genetic structure surrounding these genes. In our study, ST512 isolates (CRK2202 and CRKP2205) carried IncC plasmids with *bla*_NDM-1_ and IncFIB(pQil)/IncFII(K) plasmids with *bla*_KPC-3_. On the other hand, the ST11 isolate (BSI_389-23) carried an IncFII(pKPX1) plasmid with *bla*_NDM-1_ and an IncFIB(K)/IncFII(K) plasmid with *bla*_KPC-3_. These findings align with previous studies (17, 24, 28, 29), suggesting that *bla*_NDM-1_ may be associated with a higher diversity of plasmids (IncC or IncF in our study) than the *bla*_KPC-3_ gene and that *bla*_NDM-1_ may be co-located with *bla*_CTX-M-15_ on IncF plasmids. However, homologous regions between the plasmids of all three clones suggest possible links or movements of patients between the two hospitals.

Our analysis found that genes in KP isolates, whether located on plasmids or chromosomes, impacted several antimicrobial agents crucial for BSI. According to the Elias at al.’s study (23), the *mgrB*/*pmrB* genes conferring resistance to colistin, a last-resort treatment for MDR Gram-negative bacterial infections (4), displayed mutations across all three isolates (CRK2202, CRKP2205, and BSI_389-23). Despite suggestions of potential cross-resistance between colistin and cefiderocol (30), the colistin-resistant isolate BSI_389-23 remained phenotypically susceptible to cefiderocol. This aligns with evidence indicating that co-expression of multiple β-lactamases, often in combination with porin (OmpK35/OmpK36) mutations, may not suffice to induce cefiderocol resistance in KP isolates (30). Notably, except for BSI_389-23, which harbored the *ybt* gene, no simultaneous presence of AMR and virulence determinants was found in all isolates.

### Strengths and limitations

Our study, based on WGS analysis of a limited number of KP isolates, aligns with similar research on KPC/NDM co-producing carbapenem-resistant KP isolates from hospitalized patients (14, 31). Despite its retrospective nature and limitations in drawing firm conclusions on isolate clustering due to the lack of detailed patient movement and environmental culture data, our findings are relevant for strengthening strategies against the dissemination of MDR organisms in Italian hospitals. To address the global concern of plasmid-mediated carbapenem resistance gene spread, we employed LR WGS on 50% of isolates, chosen based on core-genome clustering. Similar strategies have been used in related studies (32). Recognizing the limitations of Kleborate analysis (21, 24), we supplemented it with AMRFinderPlus for a more comprehensive characterization of AMR and virulence determinants from genome data.

### Conclusions

Our findings enhance understanding of NDM/KPC co-producing KP within CG258 (ST512 and ST11), which may display a BSI-associated MDR phenotype. WGS analysis revealed plasmid localization of most AMR genes, including *bla*_KPC-3_ and *bla*_NDM-1_, with strong evidence of clonal transmission among patients. This emphasizes the critical role of genomic surveillance in mitigating the emergence and spread of AMR genes or plasmids associated with these isolates in hospital settings.

## MATERIALS AND METHODS

### Study isolates

Of KP isolates included in the study, five (CRKP2201 to CRKP2205) were from BSIs that occurred in COVID-19 patients at the GVM–ICC COVID-3 hospital in Rome, Italy, during a 6-month period (February to August) in 2022. A sixth isolate (BSI_389-23) was from BSI that occurred in a non-COVID-19 patient at the Fondazione Policlinico Universitario A. Gemelli IRCCS hospital in Rome, Italy, in February 2023. Isolates were identified at the species level using the Bruker Daltonics (Bremen, Germany) Biotyper matrix-assisted laser desorption ionization-time of flight mass spectrometry system.

The susceptibility of the isolates to all clinically relevant antimicrobial agents was tested using broth microdilution or disk diffusion methods (Table S1), and the minimum inhibitory concentration results were interpreted according to the European Committee on Antimicrobial Susceptibility Testing 13.0 version breakpoints (https://www.eucast.org/fileadmin/src/media/PDFs/EUCAST_files/Breakpoint_tables/v_13.0_Breakpoint_Tables.pdf). Among β-lactams, imipenem and meropenem had a minimum inhibitory concentration of >8 µg/mL. All isolates were found to co-produce NDM- and KPC-type carbapenemases using the NG-Test Carba 5 immunochromatographic assay (NG Biotech, Guipry, France) and to exhibit resistance to CAZavi.

Five patients died within 3–6 days after BSI onset (i.e., when diagnostic blood cultures were taken and patients were empirically treated with broad-spectrum antibiotics), while the sixth patient (i.e., infected with the BSI_389-23 isolate) survived and received cefiderocol as antimicrobial therapy.

### DNA extraction and whole-genome sequencing

Before sequencing, KP isolates (*n* = 6) were grown overnight at 37°C on 5% sheep blood agar and subjected to DNA extraction using the DNeasy Blood and Tissue kit (QIAGEN, Hilden, Germany) protocol. The concentration and purity of extracted DNA were determined using a NanoDrop2000 spectrophotometer (Thermo Fisher, Waltham, MA).

DNA libraries for Illumina sequencing were prepared with the Nextera Flex kit (Illumina, San Diego, CA) according to the manufacturer’s recommendations, followed by paired-end SR sequencing on an Illumina NovaSeq 6000 platform (Illumina, San Diego, CA). Sequences were pre-processed, quality controlled, and deduplicated using fastp (v.0.23.2) (33) and were then assembled using Shovill (v.1.1.0; https://github.com/tseemann/shovill) with SPAdes (v3.14.1) (34).

Three representative isolates of the clusters identified by SR cgMLST analysis (see below) were subjected to LR sequencing, which was performed using an Oxford Nanopore MinION instrument and a specific protocol (sequencing/sequencing_MIN106_DNA:FLO-MIN106:SQK-LSK109). The resulting sequences underwent base calling and trimming using guppy (v.6.0.7+c7819bc; https://nanoporetech.com/) with HAC model (template_r9.4.1_450bps_hac).

### Phylogenetic analysis

SR assemblies allowed us to determine the ST of each isolate using a seven-loci-based MLST scheme (35) and to create an *ad hoc* cgMLST scheme using chewBBACA (v.2.6.0) (36). This resulted in a scheme of 864 loci, based only on genes present in 100% of the genomes that we analyzed. A minimum spanning tree was generated from the cgMLST scheme profiles and visualized using GrapeTree (v.1.5.0) (37).

SR assemblies were mapped to the BSI_389-23 isolate’s Unicycler hybrid assembly using Snippy (v.4.6.0; https://github.com/tseemann/snippy). After detecting phage sites for BSI_389-23 masking using phispy (v.4.2.21) (38), core-genome SNPs were identified using snippy-core (with the “--mask” parameter and the output from phispy). Recombinant regions were also masked using gubbins (v.2.4.1) (39) and SNP-sites (v.2.3.3) (40). A minimum spanning tree was generated from the core-genome SNPs and visualized using the GrapeTree as mentioned above.

### Identification of plasmids

SR and LR sequences were hybrid assembled using Unicycler (v.0.5.0) (41), and the hybrid assembly of plasmids was performed using plassembler (v.1.0.0; https://github.com/gbouras13/plassembler). Plassembler hybrid assemblies were reoriented on the *repA* gene using dnaapler (v.0.1.0; https://github.com/gbouras13/dnaapler). Unicycler and plassembler hybrid assemblies were annotated with Bakta (v.1.8.1) (42) and visualized using Bandage (v.0.8.1) (43).

Unicycler hybrid assemblies were analyzed using mob-suite (v.3.0.0) (44, 45), and the analysis output was validated by comparing both Unicycler/mob-suite and plassembler results. Plasmids shared between the cluster-representative isolates were identified using a combination of mash distance (Mashtree; v.1.2.0) (46) and shared AMR genes, followed by alignment using MUMmer (v.4) (47) and visualization using pyGenomeViz (v.0.3.2; https://github.com/moshi4/pyGenomeViz). Mash distances were calculated from assembled contigs and were used as a surrogate of the average nucleotide identity (Fig. S1), which is a common measure of genome similarity.

### Identification of AMR or virulence determinants

The SR assemblies or Unicycler hybrid assemblies mentioned above were analyzed using Kleborate (v.2.3.2) (21) and AMRFinderPlus (v. 3.11.11–2023-04-17.1) (48). The Unicycler hybrid assemblies were also used to identify the sequences of the colistin resistance genes *mgrB* and *pmrB* (https://github.com/klebgenomics/Kleborate/blob/main/kleborate/data/MgrB_and_PmrB.fasta), which were analyzed at both nucleotide (using BLASTn) and amino acid (using tBLASTx) levels by means of BLAST (v.2.14.0) (49). Identified *mgrB* and *pmrB* genes were extracted using the subcommand “subseq” from seqkit (v2.4.0) (50), and multiple sequence alignments were generated using MAFFT (v.7.502) (51) and then visualized using pymsaviz (v.0.4.0; https://github.com/moshi4/pyMSAviz).

## Data Availability

All raw sequence data have been uploaded to the NCBI Sequence Read Archive Database (Bioproject accession number: PRJNA942324).

## SUPPLEMENTAL MATERIAL

Figure S1 spectrum.03305-23-s0001.pdf Heatmap of mash distances between assembled contigs for the plasmids identified in KP isolates CRKP2202, CRKP2205, and BSI_389-23. Distances are expressed as color gradient values between 0.00 to 0.10. Values in the darker range of the gradient indicate greater similarity.

Table S1 spectrum.03305-23-s0002.pdf Antimicrobial susceptibility testing (AST) results for six *Klebsiella pneumoniae* isolates included in the study.

ASM does not own the copyrights to Supplemental Material that may be linked to, or accessed through, an article. The authors have granted ASM a non-exclusive, world-wide license to publish the Supplemental Material files. Please contact the corresponding author directly for reuse.
