# Supplementary material for: In-depth characterization of multidrug-resistant NDM-1 and KPC-3 co-producing Klebsiella pneumoniae bloodstream isolates from Italian hospital patients
Source: Microbiol Spectr. 2024 Feb 27;12(4):e03305-23. doi: 10.1128/spectrum.03305-23 (PMC10986569; doi:10.1128/spectrum.03305-23)
Supplement: Figure S1 — Heatmap of mash distances between assembled contigs for the plasmids identified in KP isolates CRKP2202, CRKP2205, and BSI_389-23. Distances are expressed as color gradient values between 0.00 to 0.10. Values in the darker range of the gradient indicate greater similarity. [file spectrum.03305-23-s0001.pdf]

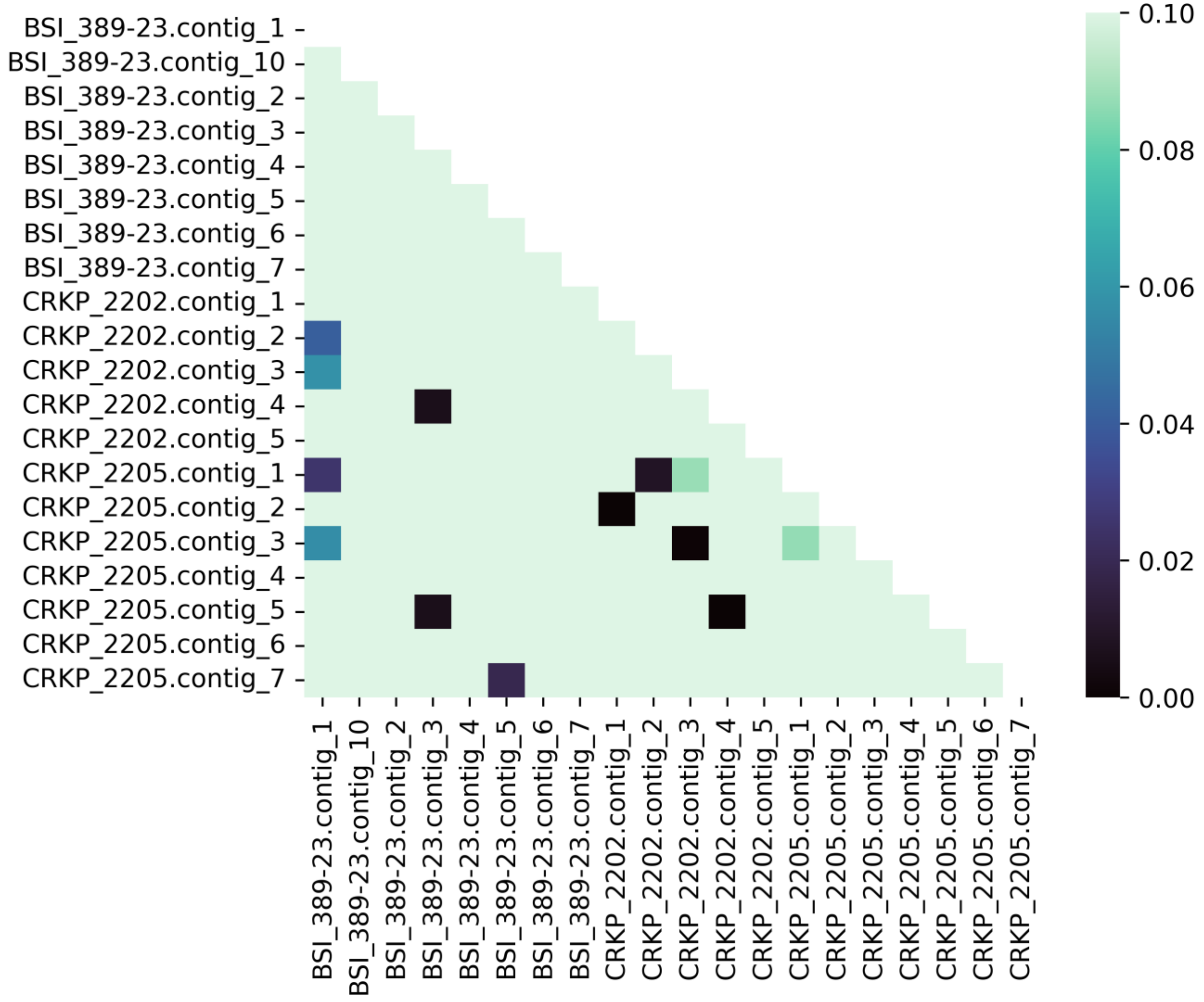

FIG. S1. Heatmap of mash distances between assembled contigs for the plasmids identified in KP isolates CRKP2202, CRKP2205, and BSI_389-23. Distances are expressed as color gradient values between 0.00 to 0.10. Values in the darker range of the gradient indicate greater similarity.
