# Supplementary material for: In-depth characterization of multidrug-resistant NDM-1 and KPC-3 co-producing Klebsiella pneumoniae bloodstream isolates from Italian hospital patients
Source: Microbiol Spectr. 2024 Feb 27;12(4):e03305-23. doi: 10.1128/spectrum.03305-23 (PMC10986569; doi:10.1128/spectrum.03305-23)
Supplement: Table S1 — Antimicrobial susceptibility testing (AST) results for six Klebsiella pneumoniae isolates included in the study. [file spectrum.03305-23-s0002.pdf]

TABLE S1 Antimicrobial susceptibility testing (AST) results for six *Klebsiella pneumoniae* isolates included in the study[a]

Class and antimicrobial agent	AST method	AST result, expressed as minimum inhibitory concentration (μg/ml) or inhibition zone diameter (mm), with interpretive category indicated in parenthesis, for the isolate					
		CRKP2201	CRKP2202	CRKP2203	CRKP2204	CRKP2205	BSI_329-23
β-lactams							
Amoxicillin-clavulanate	Vitek 2 BMD	>16 (R)	>16 (R)	>16 (R)	>16 (R)	>16 (R)	>16 (R)
	Micronaut BMD	>64 (R)	>64 (R)	>64 (R)	>64 (R)	>64 (R)	>64 (R)
Cefepime	Vitek 2 BMD	>16 (R)	>16 (R)	>16 (R)	>16 (R)	>16 (R)	>16 (R)
	Micronaut BMD	>16 (R)	>16 (R)	>16 (R)	>16 (R)	>16 (R)	>16 (R)
Cefiderocol	Disk diffusion	0 (R)	10 (R)	0 (R)	0 (R)	0 (R)	23 (S)
Cefotaxime	Vitek 2 BMD	>32 (R)	>32 (R)	>32 (R)	>32 (R)	>32 (R)	>32 (R)
Ceftazidime	Vitek 2 BMD	>32 (R)	>32 (R)	>32 (R)	>32 (R)	>32 (R)	>32 (R)
	Micronaut BMD	>64 (R)	>64 (R)	>64 (R)	>64 (R)	>64 (R)	>64 (R)
Ceftazidime-avibactam	Vitek 2 BMD	>8 (R)	>8 (R)	>8 (R)	>8 (R)	>8 (R)	>8 (R)
	Micronaut BMD	>64 (R)	32 (R)	>64 (R)	>64 (R)	>64 (R)	>64 (R)
Ceftolozane-tazobactam	Micronaut BMD	>64 (R)	>64 (R)	>64 (R)	>64 (R)	>64 (R)	>64 (R)
Ertapenem	Micronaut BMD	>2 (R)	>2 (R)	>2 (R)	>2 (R)	>2 (R)	>2 (R)
Imipenem	Vitek 2 BMD	>8 (R)	>8 (R)	>8 (R)	>8 (R)	>8 (R)	>8 (R)
Meropenem	Vitek 2 BMD	>8 (R)	>8 (R)	>8 (R)	>8 (R)	>8 (R)	>8 (R)
	Micronaut BMD	>64 (R)	>64 (R)	>64 (R)	>64 (R)	>64 (R)	>64 (R)
Piperacillin-tazobactam	Vitek 2 BMD	>64 (R)	>64 (R)	>64 (R)	>64 (R)	>64 (R)	>64 (R)
	Micronaut BMD	>128 (R)	>128 (R)	>128 (R)	>128 (R)	>128 (R)	>128 (R)
Aminoglycosides							
Amikacin	Vitek 2 BMD	32 (R)	32 (R)	32 (R)	32 (R)	32 (R)	32 (R)
	Micronaut BMD	>16 (R)	>16 (R)	>16 (R)	>16 (R)	>16 (R)	>16 (R)
Gentamicin	Vitek 2 BMD	>8 (R)	>8 (R)	>8 (R)	>8 (R)	>8 (R)	>8 (R)
	Micronaut BMD	>8 (R)	>8 (R)	>8 (R)	>8 (R)	>8 (R)	>8 (R)
Tobramycin	Vitek 2 BMD	>8 (R)	>8 (R)	>8 (R)	>8 (R)	>8 (R)	>8 (R)
Fluoroquinolones							
Ciprofloxacin	Vitek 2 BMD	>2 (R)	>2 (R)	>2 (R)	>2 (R)	>2 (R)	>2 (R)
	Micronaut BMD	>1 (R)	>1 (R)	>1 (R)	>1 (R)	>1 (R)	>1 (R)
Folate antagonists							
Trimethoprim-sulfamethoxazole	Vitek 2 BMD	>160 (R)	>160 (R)	>160 (R)	>160 (R)	>160 (R)	>160 (R)
	Micronaut BMD	>8 (R)	>8 (R)	>8 (R)	>8 (R)	>8 (R)	>8 (R)
Polymyxins							
Colistin	Micronaut BMD	>4 (R)	>4 (R)	>4 (R)	>4 (R)	>4 (R)	>4 (R)

[a]For each *K. pneumonaie* isolate, susceptibility testing to all listed antimicrobial agents, except cefiderocol, was performed with the Vitek 2 system (bioMérieux) using the AST-N397 card and/or with the Micronaut system (MERLIN Diagnostics) using ITGN microtitration plates. Both systems are current adaptations of EUCAST and CLSI reference broth microdilution (BMD) methods. The susceptibility testing to cefiderocol was performed with the disk diffusion method, using cefiderocol 30-μg disc (Liofilchem) and Mueller-Hinton E agar (bioMérieux) as per EUCAST guidelines (https://www.eucast.org/ast-of-bacteria/warnings). AST results were interpreted using EUCAST 13.0 version breakpoints (https://www.eucast.org/fileadmin/src/media/PDFs/EUCAST_files/Breakpoint_tables/v_13.0_Breakpoint_Tables.pdf), according to which all isolates were included in the resistant (R) category.
